# IBD in the Time of COVID19

**DOI:** 10.1093/crocol/otaa029

**Published:** 2020-04-16

**Authors:** Alan Moss, Miguel Regueiro

Patients and healthcare teams are facing unprecedented stresses as a result of the current severe acute respiratory syndrome coronavirus 2 (SARS-CoV-2) global pandemic. We are always conscious of the risk of infections in patients with Crohn’s disease and ulcerative colitis, but the global burden of COVID19 has raised many questions for us as clinicians. To provide some data and reference points to address these questions, we are pleased to publish a “hot topic” review by Higgins, Danese, Ng, and Rao in this issue of *CC360*. They share experience from Asia and Italy, and ongoing collaborative efforts worldwide. As data about COVID19 emerges on a daily basis, our goal is to provide a medium for rapid dissemination of peer-reviewed work on its relevance to IBD. The Open access digital format of *CC360* allows universal access to this information without any paywalls. At a time when many of us feel overwhelmed with the scope of this SARS-CoV-2 pandemic, we will continue to focus on informing our community with the most relevant information for managing IBD.

“Knowledge itself is power”—Sir Francis Bacon, *Mediationes Sacrae* (1597)

